# Die operative Therapie des benignen Prostatasyndroms in Deutschland: Eine Darstellung der Versorgungssituation auf Basis der Qualitätsberichte von 2006 bis 2019

**DOI:** 10.1007/s00120-022-01777-9

**Published:** 2022-02-16

**Authors:** Annemarie Uhlig, Martin Baunacke, Christer Groeben, Angelika Borkowetz, Björn Volkmer, Sascha A. Ahyai, Lutz Trojan, Nicole Eisenmenger, Andreas Schneider, Christian Thomas, Johannes Huber, Marianne Leitsmann

**Affiliations:** 1 Klinik für Urologie, Universitätsmedizin Göttingen, Georg-August-Universität, Robert-Koch-Straße 40, 37075 Göttingen, Deutschland; 2 Klinik und Poliklinik für Urologie, Medizinische Fakultät Carl Gustav Carus, TU Dresden, Dresden, Deutschland; 3 Klinik für Urologie, Universitätsklinikum Marburg, Philipps-Universität Marburg, Marburg, Deutschland; 4 Klinik für Urologie, Klinikum Kassel, Kassel, Deutschland; 5 Universitätsklinik für Urologie, LKH-Univ. Klinikum Graz, Medizinische Universität Graz, Graz, Österreich; 6 Reimbursement Institute, Hürth, Deutschland; 7 Gemeinschaftspraxis für Urologie, Buchholz, Deutschland

**Keywords:** Benigne Prostatahyperplasie, Benigne Prostataobstruktion, Therapieoptionen, Transurethrale Prostataresektion, Adenomenukleation, Benign prostatic hyperplasia, Benign prostatic obstruction, Therapeutic options, Transurethral resection of the prostate, Enucleation for prostate adenoma

## Abstract

**Hintergrund:**

Die operative Therapie des benignen Prostatatsyndroms (BPS) hat in den letzten Jahren an Diversität gewonnen.

**Ziel der Arbeit:**

Ziel dieser Studie ist die Darstellung aktueller Therapietrends sowie der Versorgungssituation in Deutschland.

**Material und Methoden:**

Auf Basis der Qualitätsberichte der Krankenhäuser wurden mithilfe der Onlineplattform reimbursement.INFO Diagnose- wie Eingriffszahlen erhoben. Für die benigne Prostatahyperplasie (BPH) wurden die ICD-Codes N40 und D29.1 ausgewertet. Die Prozeduren wurden mittels der OPS-Codes 5‑600.0, 5‑601, 5‑603, 5‑609.4 und 5‑609.8 inklusive Subcodierungen extrahiert. Es erfolgten eine deskriptive Darstellung, Trend- und Korrelationsanalysen.

**Ergebnisse:**

Insgesamt wurden 2019 83.687 BPS-Operationen in 473 urologischen Fachabteilungen durchgeführt. Am häufigsten wurde die transurethrale Prostataresektion (TURP; 71,7 %) angewendet. Die Holmiumlaserenukleation (HoLEP; 9,5 %) bzw. die chirurgische Adenomektomie (5,6 %) waren das zweit- bzw. dritthäufigste Verfahren. Seltener wurden Thuliumlaserenukleation (ThuLEP; 3,1 %), Laservaporisation (2,9 %) und elektrische Vaporisation (2,8 %) durchgeführt. Alle weiteren Verfahren machten jeweils < 1 % aus. HoLEP, ThuLEP und elektrische Vaporisation erlebten seit 2006 eine stetige Zunahme der Eingriffszahlen (HoLEP: +42,42 %/Jahr; *p* < 0,001, ThuLEP: +20,6 %/Jahr, *p* = 0,99; elektrische Vaporisation: +43,42 %/Jahr, *p* < 0,001), während die chirurgische Adenomektomie abnahm (−1,66 %/Jahr, *p* < 0,001). Die Krankenhausverweildauer lag 2019 bei mittleren 5,1 ± 0,1 Tagen.

**Schlussfolgerung:**

Die TURP bleibt das am häufigsten durchgeführte Operationsverfahren. Während, insbesondere in Zentren, die Lasertherapien zunehmen, geht die chirurgische Adenomektomie zurück.

**Zusatzmaterial online:**

Die Online Version dieses Artikels (10.1007/s00120-022-01777-9) enthält weitere Tabellen und Abbildungen zur Entwicklung der Eingriffszahlen für die chirurgische Adenomresektion, Urologische Fachabteilungen mit den höchsten Eingriffszahlen, Entwicklung der Eingriffe TURP, chirurgische Adenomektomie, HoLEP und ThuLEP in den 5 Häusern mit den meisten BPS-Eingriffen und zur Deutschlandweiten Verteilung der BPH-Diagnosen.

## Abstract (en)

**Background:**

The surgical management of benign prostatic obstruction (BPO) has greatly evolved in recent years.

**Objectives:**

The aim of this study is to present contemporary management and trends for surgical BPO therapy in Germany.

**Materials and methods:**

Disease and procedure rates were extracted using the online platform reimbursement.INFO that is based on German hospital quality report data. For the diagnosis of benign prostate hyperplasia (BPH), the ICD codes N40 and D29.1 were used. For evaluation of the surgical procedures OPS codes 5‑600.0, 5‑601, 5‑603, 5‑609.4 and 5‑609.8 including their subcodes were used. In addition to descriptive analyses, trend and correlation analyses were performed.

**Results:**

In 2019, a total of 83,687 procedures for BPO in 473 urological departments were performed. The most common (71.7%) surgery was transurethral resection of the prostate (TUR-P). Holmium laser enucleation of the prostate (HoLEP; 9.5%) and surgical adenomectomy (5.6%) were the second and third most common procedures. Less often thulium laser enucleation (ThuLEP; 3.1%), laser vaporisation (2.9%) and electrical vaporisation (2.8%) were performed. All other techniques were performed in < 1%. Rates of HoLEP, ThuLEP and electrovaporisation have increased since 2006 (HoLEP: +42.42%/year, *p* < 0.001; ThuLEP: +20.6%/year, *p* = 0.99; electrovaporisation +43.42%/year, *p* < 0.001), while surgical adenomectomy decreased (−1.66%/year, *p* < 0.01). In 2019 mean length of hospital stay was 5.1 ± 0.1 days.

**Conclusions:**

TUR‑P remains the most often performed surgical treatment for BPO. Laser therapy—especially in centers—is increasing, while surgical adenomectomy continues to abate.

## Hintergrund und Fragestellung

Das benigne Prostatasyndrom (BPS) kann mit ca. 5 Mio. betroffenen Männern in Deutschland als „Volkskrankheit“ bezeichnet werden [15]; seine Prävalenz steigt mit zunehmendem Lebensalter kontinuierlich an. Therapieziele in der Behandlung des BPS sind die Wiederherstellung von Lebensqualität sowie der Funktionalität des unteren Harntraktes. Medikamentöse Therapien können Symptome oftmals lindern und eine operative Therapie hinauszögern oder gänzlich unnötig machen [16]. Trotzdem muss sich im Verlauf jeder 5. von einem BPS betroffene Mann einer operativen Therapie unterziehen [8].

Die transurethrale Resektion der Prostata (TURP) bildet seit Jahrzehnen den Referenzstandard der operativen endoskopischen Therapie bei einer mittleren Prostatagröße [8]. Jedoch hat sich die Landschaft der operativen Möglichkeiten in den letzten Jahren stark erweitert und v. a. durch den Einsatz der Lasertherapien an Diversität gewonnen [17]. Bei der Entwicklung operativer Verfahren lag historisch der Fokus auf möglichst sicheren Therapieoptionen mit geringem Risiko für ein TUR-Syndrom sowie Blutungen bzw. Transfusionen und einem kurzen Krankenhausaufenthalt. Mittlerweile besteht der Anspruch darin, neben der Sicherheit der Verfahren, funktionell bessere Ergebnisse bezüglich der Vermeidung einer retrograden Ejakulation, erektiler Dysfunktion oder Harninkontinenz im Vergleich zur bewährten TURP zu erzielen [17]. Die aktuelle Leitlinie der European Association of Urology (EAU) empfiehlt beispielsweise die Laservaporisation mittels Kaliumtitanylphosphat- (KTP-) oder Lithiumtriborat- (LBO-)Laser als Alternative zur TURP sowie die Holmiumlaserenukleation der Prostata (HoLEP) als effiziente, minimal-invasive, prostatagrößenunabhängige operative Therapie des BPS [8]. Die Gleichwertigkeit der HoLEP gegenüber der TURP hinsichtlich funktioneller Ergebnisse und teilweise sogar eine Überlegenheit in Bezug auf Komplikationen wurden in mehreren Arbeiten demonstriert [4, 5]. Neue Verfahren wie die Wasserablation (Aquablation) scheinen ebenfalls vielversprechend [7]. Dieses und andere Verfahren (z. B. Prostataarterienembolisation) haben bereits Einzug in die Leitlinien gehalten, für einige Therapien ist der Empfehlungsgrad sogar hoch (Prostata-Harnröhren-Lifting-Verfahren; [8]). Die klassische offene Adenomenukleation hingegen sollte aufgrund ihrer erhöhten Morbidität nur mehr bei fehlender Verfügbarkeit endoskopischer Verfahren oder auf Patientenwunsch angewendet werden [8].

Für die deutsche Versorgungssituation verfügbare Studienergebnisse belegen die Prädominanz der TURP bei der operativen Therapie des BPS [2, 9, 10, 13]. Inwieweit sich die Anwendung der einzelnen operativen Methoden in den letzten 15 Jahren verändert hat, ist jedoch bis dato unklar.

Ziel dieser Studie ist daher die Versorgungswirklichkeit der operativen Therapie des BPS in Deutschland darzustellen.

## Studiendesign und Untersuchungsmethoden

### Datenquelle

Datengrundlage waren Krankenhausqualitätsberichte, welche über die Onlineplattform reimbursement.INFO akquiriert wurden. Die Plattform bietet u. a. die Möglichkeit, Informationen aus den Qualitätsberichtsdaten des Gemeinsamen Bundesausschusses (Marktanalyse) sowie zu Daten des Statistischen Bundesamtes (Patientenanalyse) und des Instituts für das Entgeltsystem im Krankenhaus (G-DRG-Analyse) zu analysieren. Auch Betrachtungen auf Fachabteilungsebene (Marktanalyse) für multiple Jahrgänge sowie demographische Untersuchungen (Alter, Geschlecht und Aufenthaltsdauer – allerdings nicht auf Fachabteilungsebene) sind möglich. Aus Datenschutzgründen wird in den Qualitätsberichtsdaten für Diagnose- (ICD) bzw. Eingriffszahlen (OPS) mit einer Anzahl von ≤ 3 die tatsächliche Zahl nicht ausgewiesen, sondern die Zahl 1 angegeben.

Alle verwendeten Daten sind anonymisiert, daher war kein Ethikvotum erforderlich.

### Endpunkte

Primär wurden die Eingriffszahlen für die operativen BPS-Therapien auf Fachabteilungs- bzw. Bundesebene dargestellt. Für operative Prozeduren wurden die OPS-Codes 5‑600.0, 5‑601, 5‑603, 5‑609.4 und 5‑609.8 inklusive Subkodierungen verwendet. Die „chirurgische Adenomektomie“ umfasste sowohl die offenen als auch die laparoskopischen (roboterassistierten) Verfahren.

Nicht möglich auf reimbursement.INFO ist eine gemeinsame Betrachtung von ICD- und OPS-Codes auf Patientenebene, d. h. für einen individuellen Patienten mit der Diagnose einer benignen Prostatahyperplasie (BPH) kann die erfolgte Therapie nicht direkt ermittelt werden. Daher erfolgte eine Korrelation von ICD- und OPS-Fallzahlen auf Fachabteilungsebene. Die Ermittlung der Diagnosezahlen für die BPH erfolgte nach ICD N40.0 und D29.1.

Neben der Ermittlung der Gesamtzahlen auf Bundesebene für das Jahr 2019 erfolgte eine Untersuchung der Fallzahländerung über den Erhebungszeitraum (je nach Eingriff/Diagnose ab 2006 oder später bis 2019). Für die operativen Eingriffe wurden zudem die einzelnen Operationsverfahren in ihrer Anwendungshäufigkeit für 2019 getrennt betrachtet. Des Weiteren wurde untersucht, welche Kliniken im bundesweiten Vergleich die meisten Eingriffe durchführten bzw. die meisten Diagnosen stellten. Deutschlandweite Verteilungen von Eingriffszahlen für die 4 häufigsten BPS-Therapien sowie für die Diagnose einer BPH wurden auf Deutschlandkarten visualisiert. Die Kartendarstellungen erfolgten mittels der Software „EasyMap 11.1 Standard Edition“ (Lutum + Tappert DV-Beratung GmbH, Bonn, Deutschland).

Außerdem wurden das mittlere Patientenalter und dessen Änderung über die Jahre sowie die mittlere Aufenthaltsdauer im Jahr 2019 auf Klinikebene untersucht.

### Statistische Analyse

Bei Prozeduren basierten sämtliche Nennungen auf den Einfachkodierungen je Fall (ohne Duplikate). Kontinuierliche Parameter (Alter und Aufenthaltsdauer) wurden als Mittelwert (MW) und Standardabweichung (SD) angegeben, kategoriale Daten als Absolutwerte und Prozente. Statistische Vergleiche der Entwicklung der Altersgruppen (Kategorien) erfolgten mithilfe des χ^2^-Tests, Korrelationsanalysen mithilfe der Rangkorrelation nach Spearman. Verteilungen wurden mithilfe des Shapiro-Wilk-Tests überprüft. Die Trendentwicklung jährlicher Fallzahlen wurde mithilfe eines nichtparametrischen Test für Trends berechnet [18].

Die statistische Auswertung erfolgte mit R (R Foundation for Statistical Computing, Wien, Österreich) Version 4.0.2 und R Studio (RStudio, PBC, Boston, MA, USA) Version 1.3.1093. Das Signifikanzniveau wurde mit *p* = 0,05 definiert. Alle statistischen Tests sind zweiseitig.

## Ergebnisse

### Operative Eingriffe

**Figure 1:**
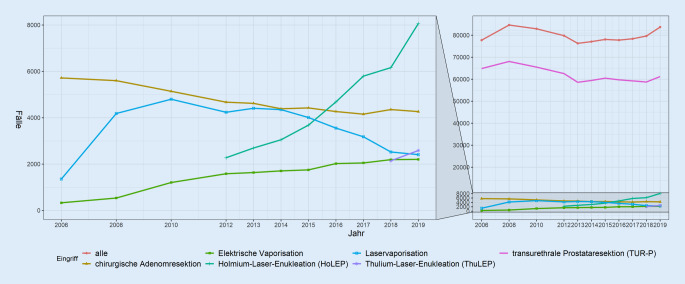

Im Jahr 2019 wurden insgesamt 83.687 BPS-Operationen in 473 urologischen Fachabteilungen durchgeführt. Im Vergleich dazu erfolgten 2006 77.747 Eingriffe in noch 478 urologischen Abteilungen. Insgesamt war, nach initial deutlicher Zunahme (2006–2008 +8,9 %), zwischen 2008 und 2013 ein Abfall der Eingriffszahlen um −9,8 % zu beobachten, gefolgt von einem erneuten Anstieg, bei dem bis 2019 die Eingriffszahlen aus 2008 knapp wieder erreicht wurden (Differenz 928 Eingriffe, 98,9 % von 2008; Abb. 1).

Das im Jahr 2019 mit Abstand am häufigsten angewendete operative BPS-Verfahren war die TURP, welche insgesamt 71,7 % aller Eingriffe ausmachte. Mit insgesamt 9,5 % war die HoLEP der zweithäufigste Eingriff, gefolgt von der chirurgischen Adenomektomie, welche 5,6 % der Eingriffe ausmachte. Seltener wurden Thuliumlaserenukleation (ThuLEP) in 3,1 %, Laservaporisation in 2,9 % und elektrische Vaporisation in 2,8 % durchgeführt. Alle weiteren Verfahren wurden anteilig zu je < 1 % durchgeführt. Eine Aufschlüsselung operativen BPS-Therapien für 2019 zeigt Tab. 1.

**Table 1:** 

Eingriff	OPS-Code	Anzahl (*n*)	Prozent (%)
*Transurethrale Prostataresektion (TURP)*	*5‑601.0; 5‑601.1*	*61.198*	*73,13*
*Holmiumlaserenukleation (HoLEP)*	*5‑601.70*	*8064*	*9,64*
*Chirurgische Adenomektomie*^a^	*5‑603*	*4266*	*5,09*
*Thuliumlaserenukleation (ThuLEP)*	*5‑601.72*	*2586*	*3,09*
*Laservaporisation*	*5‑601.42*	*2409*	*2,88*
*Elektrische Vaporisation*	*5‑601.6*	*2206*	*2,64*
Thuliumlaserresektion	5‑601.73	582	0,7
Sonstige Destruktion oder Exzision	5‑601.x,y	450	0,54
Exzision durch fokussierten Wasserstrahl (Aquablation)	5‑601.9	442	0,53
Prostatainzision	5‑600.0	401	0,48
Visuell kontrollierte laserunterstützte Ablation (VLAP)	5‑601.41	327	0,39
Wasserdampfablation	5‑601.32	317	0,38
Holmiumlaserresektion	5‑601.71	149	0,18
Sonstige Laserexzision	5‑601.7x	71	0,08
Hitzedestruktion	5‑601.33,34,3x	38	0,05
Stent	5‑609.4	38	0,05
Sonstige Laserdestruktion	5‑601.4x	35	0,04
Destruktion durch magnetresonanzgesteuerten Ultraschall	5‑601.a	29	0,03
Elektroporation	5‑601.8	26	0,03
Geweberetraktor	5‑609.80–85	26	0,03
Interstitielle Laserdestruktion	5‑601.40	11	0,01
Nadelablation (TUNA)	5‑601.5	7	0,01
Radiofrequenzablation	5‑601.30	4	< 0,01
Kryodestruktion	5‑601.2	3	< 0,01
Mikrowellenablation	5‑601.31	2	< 0,01

Kursiv: Verfahren mit einer anteiligen Anwendungshäufigkeit von > 1 %

^a^Offene und laparoskopische (roboterassistierte) Verfahren

Die Abb. 1 stellt die Entwicklung der Eingriffszahlen für die 6 häufigsten Eingriffe an urologischen Kliniken von 2006–2019 dar. Die TURP als konstant am häufigsten angewandtes Verfahren zeigte zwischen 2008 und 2012 eine leichte Abnahme (−8,1 %, *p* = 0,05), um sich zuletzt auf einem Niveau von knapp unter 60.000 Eingriffen/Jahr zu stabilisieren. HoLEP, ThuLEP und elektrische Vaporisation erlebten eine stetige Zunahme der Eingriffszahlen: auf 8102 Fälle von 2012–2019 für die HoLEP (+42,42 %/Jahr, *p* < 0,001), auf 2614 Fälle von 2018 bis 2019 für die ThuLEP (+20,6 %, *p* = 0,99) und auf 2376 Fälle von 2006 bis 2019 für die elektrische Vaporisation (+43,42 %/Jahr, *p* < 0,001). Die durchgeführten Laservaporisationen zeigten einen Gipfel im Jahr 2010 und ebbten hiernach wieder ab. Seit 2006 sanken die Zahlen für die chirurgische Adenomektomie (von 6130 auf 4806 Fälle, −1,66 %/Jahr, *p* < 0,001). Dieses Verfahren war das am dritthäufigsten angewendete. Von den 2019 durchgeführten 4806 chirurgischen Adenomresektionen wurden 418 (7 %) laparoskopisch bzw. roboterassistiert durchgeführt, wobei jedoch nicht explizit zwischen beiden Operationsmethoden unterschieden werden konnte (Abb. A, Zusatzmaterial online).

**Figure 2:**
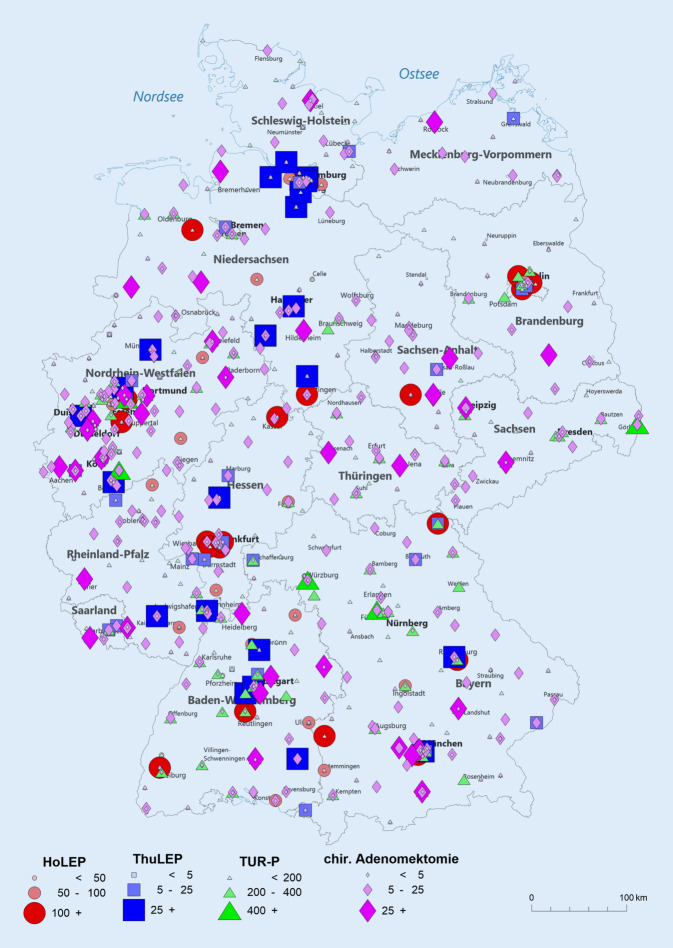

Die deutschlandweite Verteilung der 4 häufigsten operativen BPS-Therapien in deutschen urologischen Fachabteilungen für das Jahr 2019 ist in Abb. 2 dargestellt. HoLEP und ThuLEP zeigen eine deutliche Zentrenbildung. Für die ThuLEP besteht ein deutliches Gefälle zwischen alten und neuen Bundesländern. Die chirurgische Adenomektomie ist ubiquitär vertreten. Für die TURP scheint ein Gefälle von Süd- nach Norddeutschland zu bestehen.

Die Tab. A im Zusatzmaterial online zeigt die 20 urologischen Fachabteilungen mit den höchsten Eingriffszahlen im Jahr 2019. An der Klinik mit den höchsten Eingriffszahlen wurden 1,5 % aller BPS-Operationen durchgeführt. Sämtliche weiteren Kliniken hatten einen Eingriffsanteil von je < 1 %.

Die Tab. B im Zusatzmaterial online zeigt die Entwicklung der Eingriffe TURP, chirurgische Adenomektomie, HoLEP und ThuLEP in den 5 Häusern mit den meisten BPS-Eingriffen 2019 über die Jahre (2006–2019). Von 5 Kliniken bieten 3 alle 4 bundesweit am häufigsten durchgeführten Eingriffe an.

Die Daten zeigten weiterhin, dass 2019 die Fachabteilungen mit den 20 meisten Eingriffen, für jedes Verfahren separat betrachtet, vorwiegend zu nicht-universitären Häusern gehörten: TURP (19 nicht-universitäre Häuser; 95 %), chirurgische Adenomektomie (17; 85 %), HoLEP (14; 70 %) und ThuLEP (15; 75 %).

### Patienten

**Figure 3:**
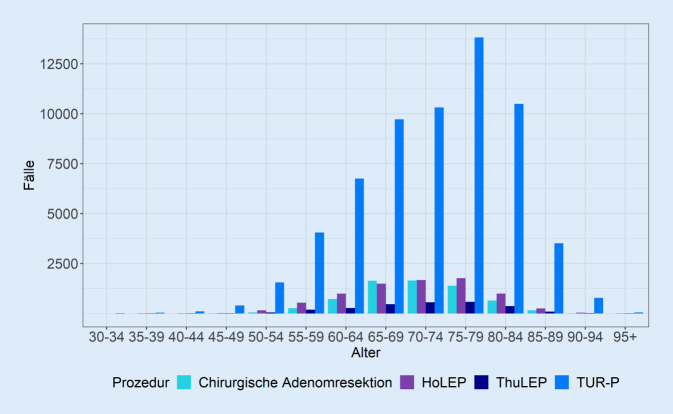

Das mittlere Patientenalter zum Zeitpunkt der BPS-Therapie lag 2019 bei ca. 72 Jahren, für die chirurgische Adenomektomie bei 72,3 Jahren, TURP 72,3 Jahre, HoLEP 71,2 Jahre und ThuLEP 71,7 Jahre (Abb. 3). Nur für einen Vergleich des mittleren Patientenalters zwischen chirurgischer Adenomektomie und TURP zeigte sich kein statistisch signifikanter Unterschied (*p* = 0,586; alle übrigen *p* < 0,001).

Die Betrachtung der Altersentwicklung der Patienten über die Jahre 2006 bis 2019 ergab für die TURP eine Zunahme des Alters um 1,5 Jahre von 70,8 auf 72,3 Jahre. Alle 4 untersuchten Eingriffe zeigte sich über die Jahre eine statistisch signifikante Zunahme des Durchschnittsalters (jeweils *p* < 0,001).

**Figure 4:**
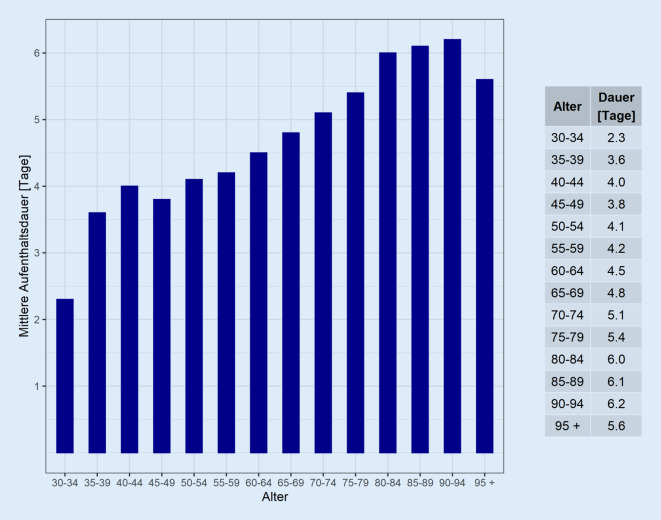

Da für operative Prozeduren keine Daten zur Aufenthaltsdauer zur Verfügung standen, wurden hierfür Daten der Patientenanalyse von BPH-Patienten untersucht. Die mittlere stationäre Aufenthaltsdauer von BPH-Patienten älter als 30 Jahre betrug 2019 5,1 ± 0,1 Tage. Mit zunehmendem Patientenalter war ein kontinuierlicher Anstieg der stationären Aufenthaltsdauer zu beobachten (Abb. 4; *p* *=* *0,01*). Die Korrelation von Fallzahlen für die BPH und Eingriffszahlen für sämtliche oben beschriebenen Eingriffe für alle urologischen Fachabteilungen für das Jahr 2019 ergab eine starke und signifikante Korrelation (Spearman’s rho: 0,993, *p* < 0,001).

Eine Darstellung zur deutschlandweiten Verteilung der BPH-Fälle findet sich im Zusatzmaterial online (Abb. B).

## Diskussion

Unsere Studie zeigt die Versorgungswirklichkeit der operativen BPS-Therapie in Deutschland zwischen 2006 und 2019. In Deutschland werden aktuell pro Jahr ca. 84.000 BPS-Operationen durchgeführt. Insgesamt zeigte sich zwischen 2008 und 2013 ein leichter Rückgang der Eingriffszahlen, welche jedoch bis 2019 fast wieder das Vorniveau erreichten. Die Beobachtung einer Abnahme für denselben Zeitraum deckt sich mit einem vorangegangenen Bericht von Leicht et al., welche diesen Rückgang mit zunehmenden medikamentösen Behandlungsmöglichkeiten begründeten [13]. Der stetige Anstieg des Durchschnittsalters der Patienten unserer Kohorte (von 70,8 auf 72,3 Jahre) könnte ebenfalls auf ein Hinauszögern der operativen Therapie durch optimierte medikamentöse Optionen schließen lassen. Auch mögen technische Verbesserungen und die Sicherheit der operativen Eingriffsmöglichkeiten dazu geführt haben, dass zunehmend ältere Patienten operiert werden.

## TURP bleibt „Goldstandard“

Die Therapielandschaft in Deutschland bildet das vielseitige Spektrum operativer Verfahren der BPS-Therapie ab. Neben der seit Jahrzehnten unbestrittenen Goldstandardoperation TURP nimmt die Anwendung alternativer Verfahren stetig zu [2]. Dies liegt nicht zuletzt an mittlerweile fundierten Langzeiterfahrungen mit jüngeren Techniken. Doch auch wenn der Anteil der TURP in den letzten Jahren von 90 % auf knapp 72 % zurückgegangen ist, wird diese nach wie vor mehr als siebenmal so oft angewendet wie die am zweithäufigsten vertretene HoLEP [3].

## Laserverfahren werden zunehmend angewendet

Insbesondere die Laserverfahren wie HoLEP und ThuLEP erlebten seit 2006 ein stetiges Wachstum der Eingriffszahlen. So hat sich die HoLEP in Deutschland zur zweithäufigsten BPS-Operation entwickelt (9,5 %), die ThuLEP liegt auf Platz 4 (3,1 %). Laserenkuleationen haben sich als sehr effiziente und v. a. sichere Verfahren etabliert und werden, aufgrund der besseren Hämostase, auch bei Patienten unter Antikoagulationsbehandlung eingesetzt [8]. Die Verfügbarkeit von Laserverfahren für große Prostatadrüsen macht ein offen-operatives Vorgehen zunehmend obsolet [11]. Trotzdem ist die chirurgische Adenomektomie in Deutschland weiterhin das am dritthäufigsten angewendete Verfahren. Diese Erkenntnis deckt sich mit einer Umfrage zur Durchführung operativer BPS-Therapien unter amerikanischen Urologen aus dem Jahr 2012: von 12 abgefragten Techniken wurden v. a. die offene Operation (78 %) und die TURP (73 %) angewendet [12].

Gute Sicherheitsprofile und funktionelle Ergebnisse sowie kürzere Liegedauern, geringere Komplikations- wie Reinterventionsraten und schlussendlich die Wiederverwendbarkeit von Materialen (z. B. Laserfaser) können die hohen Anschaffungskosten neuer Techniken rechtfertigen.

## Größte Fallzahlen auch an nicht-universitären Zentren

Die Fachabteilungen mit den größten Eingriffszahlen für TURP, HoLEP, ThuLEP und die chirurgische Adenomektomie finden sich für vorwiegend an nicht-universitären Zentren; dies gilt besonders für die schon lange etablierten Verfahren wie die TURP und die chirurgische Adenomektomie. Jedoch zeigt ein Anteil von 70 % und 75 % nicht-universitärer Fachabteilungen für HoLEP und ThuLEP, dass diese Verfahren längst über den experimentellen Charakter hinausgewachsen sind.

## „Maßgeschneiderte“ Therapieoptionen

Die Wahl der operativen Methode zur BPS-Therapie wird bestimmt von klinischen Ergebnissen, Sicherheit in der Anwendung und minimaler Invasivität [12]. Abhängig von Ausprägung der klinischen Symptomatik, Prostatagröße, Patientenalter, Komorbiditäten und Patientenwünschen ermöglichen die neuen Verfahren eine individualisierte BPS-Therapie. So hat sich der Einsatz von Lasertherapien v. a. bei großem Prostatavolumen als sichere Alternative zu TURP und chirurgischer Adenomektomie bewährt. Durch weniger invasive Therapien (z. B. Rezum) kann sexuell aktiven Männern mit großer Wahrscheinlichkeit ein Erhalt der antegraden Ejakulation ermöglicht werden [14]. Für multimorbide Patienten mit großem Narkoserisiko stehen Verfahren zur Verfügung die teils unter lokaler Anästhesie erfolgen können (z. B. UroLift und andere Geweberetraktoren oder Prostataarterienembolisation). Auch die Möglichkeit ambulant durchführbarerer Operationen ist von zunehmendem Interesse. Doch nicht nur die unmittelbaren Vorteile sondern auch Langzeitreinterventionsraten spielen eine bedeutende Rolle in der Verfahrenswahl der operativen BPS-Therapie [6].

Entsprechend benötigt eine gut aufgestellte urologische Abteilung heutzutage Alternativen zur TURP, um wettbewerbsfähig zu bleiben. Die Vielfalt der verfügbaren Eingriffe erfordert eine entsprechend komplexere Patientenberatung; für Zuweiser wie für die operativen Zentren. Studien zeigen, dass die einzelnen Verfahren unterschiedliche Anforderungen an den Operateur stellen: im Erlernen neuer Techniken sind zwar Kenntnisse anderer Methoden von Vorteil, dennoch unterscheiden sich diese teilweise deutlich voneinander und benötigen eine intensive Geräteeinweisung sowie ein strukturiertes Training [19].

## Limitationen und Stärken

Trotz ihrer Größe und der damit möglichen umfangreichen Auswertungen weist unsere Studie Limitationen auf: Erstens fehlen in der Datenbank klinische Daten, sodass weder Indikation noch Versorgungsqualität der Therapien näher charakterisiert werden können. Eine direkte Verknüpfung von ICD- und OPS-Codes ist nicht möglich. So konnte z. B. nicht festgestellt werden, ob eine BPH oder, sehr viel seltener, ein Prostatakarzinom die Indikation für eine TURP war. Daher wurden in den Analysen indirekte Vergleiche mittels Korrelationen von ICD- und OPS-Codes auf Fachabteilungsebene angestrengt. Zweitens verzerren mögliche Doppelkodierungen die Ergebnisse: Bei den kombinierten Verfahren (z. B. Aquablation) wird häufig am Ende der Operation eine Blutstillung durchgeführt und diese dann als TURP kodiert was zu einer Überschätzung der Fallzahlen führt. Dem hingegen führt die Zensierung kleiner Fall- bzw. Eingriffszahlen (d. h. die Mittelung der Eingriffszahlen ≤ 3 auf 1) zu einer Unterschätzung. Viertens lassen sich Fehlkodierungen nicht ausschließen. Bei den ICD-Codes sind sogar Umkodierungen der Hauptdiagnose BPH der Codes N40 oder D29.1 zu C61 zu erwarten, wenn sich in der Histologie ein inzidentelles Prostatakarzinom findet. Dies ist in ca. 5–14 % der Fälle möglich und dürfte somit zu einer Unterschätzung der Patientenzahlen geführt haben [1]. Fünftens konnten Zahlen zur Prostataarterienembolisation aufgrund des fehlenden selektiven OPS-Codes nicht dargestellt werden.

Trotz der genannten Limitationen ist dies die erste Studie, welche die operativen Therapieoptionen des BPS anhand von Routinedaten aufbereitet und Entwicklungen zu neueren Verfahren in Deutschland abbildet. Im Vergleich zu den Ergebnissen der Qualitätssicherung mit Routinedaten (QSR-Daten) der AOK sind hier sämtliche bundesweit verfügbaren Fälle bzw. Eingriffe unabhängig vom Versicherungsstatus der Patienten abgebildet.

## Schlussfolgerung

Die Studie zeigt, dass die TURP weiterhin das am häufigsten durchgeführte Verfahren zur operativen BPS-Therapie ist. Jedoch haben sich in den letzten Jahren viele alternative Eingriffsmethoden etabliert. In deren Verbreitung zeigen sich deutliche regionale Unterschiede. Insbesondere in Zentren nehmen die Lasertherapien zu, für die chirurgische Adenomektomie zeigen sich regrediente Eingriffszahlen.

## Fazit für die Praxis

- Die operative BPS-Therapie (benignes Prostatasyndrom) in Deutschland hat in den letzten Jahren stetig an Diversität gewonnen. Für die verschiedenen verfügbaren Verfahren zeigen sich deutliche regionale Unterschiede.
- Die transurethrale Prostataresektion (TURP) ist weiterhin das mit Abstand am häufigsten durchgeführte Operationsverfahren und macht insgesamt ca. 71 % aller Eingriffe aus.
- Lasertherapien wie Holmiumlaserenukleation der Prostata (HoLEP) und Thuliumlaserenukleation der Prostata (ThuLEP) liegen mit 9,5 % und 3,1 % auf Platz 2 und 4 der häufigsten Eingriffe und erleben seit 2006, insbesondere in Zentren, ein stetiges Wachstum der Eingriffszahlen.
- Trotz der Abnahme der durchgeführten chirurgischen Adenomektomien bleibt dieses Verfahren mit 5,6 % das am dritthäufigsten angewendete.
- Das mittlere Patientenalter zum Zeitpunkt der BPS-Therapie lag 2019 bei ca. 72 Jahren und unterscheidet sich zwischen den Verfahren kaum. Die stationäre Aufenthaltsdauer von Patienten, die sich einer operativen BPS-Therapie unterziehen, steigt mit zunehmendem Alter.

## Supplementary Information







