# Supplementary material for: Contemporary surgical management of benign prostatic obstruction in Germany: A population-wide study based on German hospital quality report data from 2006 to 2019
Source: Urologe A. 2022 Feb 16;61(5):508–17. [Article in German] doi: 10.1007/s00120-022-01777-9 (PMC9072522; doi:10.1007/s00120-022-01777-9)
Supplement: Supplementary file 1 [file 120_2022_1777_MOESM1_ESM.docx]

**Appendix**

**Abbildung A**

*
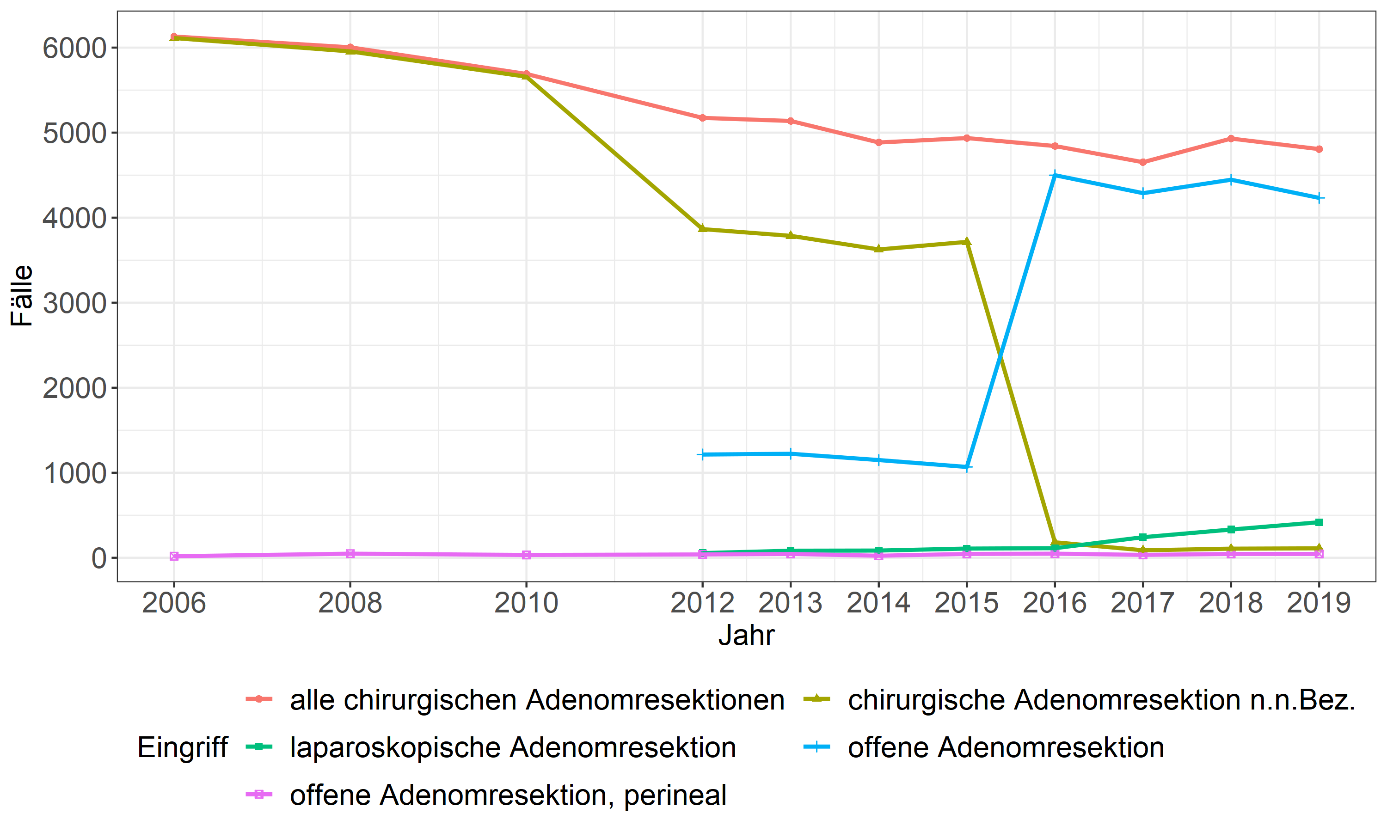
*

*Abbildung A: Entwicklung der Eingriffszahlen für die chirurgische Adenomresektion an den urologischen Kliniken von 2006 bis 2019. Die sich kreuzenden Eingriffszahlen für die „nicht näher bezeichneten chir. Adenomresektionen“ und die „offenen Adenomresektionen“ zwischen 2015 und 2016 sind Änderungen in der Codierordnung geschuldet.*
