# Supplementary material for: Contemporary surgical management of benign prostatic obstruction in Germany: A population-wide study based on German hospital quality report data from 2006 to 2019
Source: Urologe A. 2022 Feb 16;61(5):508–17. [Article in German] doi: 10.1007/s00120-022-01777-9 (PMC9072522; doi:10.1007/s00120-022-01777-9)
Supplement: Supplementary file 2 [file 120_2022_1777_MOESM2_ESM.docx]

**Tabelle A**

*Urologische Fachabteilungen mit den höchsten Eingriffszahlen im Jahr 2019.*

| Rang | Name | PLZ | Stadt | Träger | Universitäts-klinik | n | % |
| --- | --- | --- | --- | --- | --- | --- | --- |
| 1 | Vivantes Auguste-Viktoria-Klinikum | 12157 | Berlin | öffentlich | nein | 1.276 | 1,5 |
| 2 | Augusta-Kranken-Anstalt gGmbH Bochum-Mitte | 44791 | Bochum | freigemeinnützig | nein | 823 | 1,0 |
| 3 | Caritas-Krankenhaus St. Josef | 93053 | Regensburg | freigemeinnützig | nein | 655 | 0,8 |
| 4 | Klinikum der Universität München (LMU Klinikum) | 81377 | München | öffentlich | ja | 647 | 0,8 |
| 5 | Missioklinik Würzburg | 97074 | Würzburg | freigemeinnützig | nein | 601 | 0,7 |
| 6 | Diakonie-Klinikum Stuttgart | 70176 | Stuttgart | freigemeinnützig | nein | 597 | 0,7 |
| 7 | Kreisklinikum Calw-Nagold, Kliniken Nagold | 72202 | Nagold | öffentlich | nein | 537 | 0,6 |
| 8 | Asklepios Klinikum Harburg | 21075 | Hamburg | privat | nein | 511 | 0,6 |
| 9 | Vivantes Humboldt-Klinikum | 13509 | Berlin | öffentlich | nein | 502 | 0,6 |
| 10 | Klinikum Fürth | 90766 | Fürth | öffentlich | nein | 499 | 0,6 |
| 11 | Sankt Katharinen Krankenhaus GmbH | 60389 | Frankfurt | freigemeinnützig | nein | 492 | 0,6 |
| 12 | Asklepios Klinik Barmbek | 22307 | Hamburg | privat | nein | 489 | 0,6 |
| 13 | Sana Klinikum Hof | 95032 | Hof | privat | nein | 483 | 0,6 |
| 14 | Klinikum Lippe Detmold | 32756 | Detmold | öffentlich | nein | 477 | 0,6 |
| 15 | Klinikum Oldenburg AöR | 26133 | Oldenburg | öffentlich | nein | 475 | 0,6 |
| 16 | Urologische Klinik München - Planegg | 82152 | Planegg | privat | nein | 475 | 0,6 |
| 17 | Marien Hospital Herne, Klinikum der Ruhr-Universität Bochum | 44625 | Herne | freigemeinnützig | ja | 472 | 0,6 |
| 18 | Zeisigwaldkliniken Bethanien Chemnitz | 09130 | Chemnitz | freigemeinnützig | nein | 472 | 0,6 |
| 19 | Universitätsklinikum Freiburg | 79106 | Freiburg | öffentlich | ja | 461 | 0,5 |
| 20 | Universitätsklinikum Münster | 48149 | Münster | öffentlich | ja | 455 | 0,5 |
