# Supplementary material for: Contemporary surgical management of benign prostatic obstruction in Germany: A population-wide study based on German hospital quality report data from 2006 to 2019
Source: Urologe A. 2022 Feb 16;61(5):508–17. [Article in German] doi: 10.1007/s00120-022-01777-9 (PMC9072522; doi:10.1007/s00120-022-01777-9)
Supplement: Supplementary file 3 [file 120_2022_1777_MOESM3_ESM.docx]

**Tabelle B**

*Entwicklung der Eingriffe TUR-P, chirurgische Adenomektomie, HoLEP und ThuLEP in den 5 Häusern mit den meisten BPS-Eingriffen 2019 von 2006 bis 2019. Es ist zu beachten, dass Eingriffszahlen mit ≤3 Eingriffen als 1 angegeben werden.*

|  |  | Jahr | | | | | | | | | | | | |
| --- | --- | --- | --- | --- | --- | --- | --- | --- | --- | --- | --- | --- | --- | --- |
| Klinik | **Stadt** | **Eingriff** | **2006** | **2008** | **2010** | **2012** | **2013** | **2014** | **2015** | **2016** | **2017** | **2018** | **2019** |  |
|  |  |  |  |  |  |  |  |  |  |  |  |  |  |  |
| Augusta-Kranken-Anstalt gGmbH Bochum-Mitte | Bochum | Chir. Adenomektomie | 1 | 1 | 19 | 11 | 24 | 24 | 10 | 1 | - | 1 | 9 |  |
|  |  | TUR-P | 221 | 205 | 266 | 93 | 207 | 260 | 254 | 266 | 342 | 403 | 461 |  |
|  |  | HoLEP |  |  |  | 1 | - | - | 27 | 79 | 139 | 196 | 236 |  |
|  |  | ThuLEP |  |  |  |  |  |  |  |  |  | 1 | 5 |  |
| Caritas-Krankenhaus St. Josef | Regensburg | Chir. Adenomektomie | 47 | 43 | 47 | 50 | 56 | 52 | 66 | 25 | 6 | 6 | 6 |  |
|  |  | TUR-P | 318 | 284 | 278 | 312 | 327 | 372 | 367 | 368 | 311 | 293 | 293 |  |
|  |  | HoLEP |  |  |  |  |  |  |  | 38 | 197 | 263 | 350 |  |
|  |  | ThuLEP |  |  |  |  |  |  |  |  |  |  | - |  |
| Diakonie-Klinikum Stuttgart | Stuttgart | Chir. Adenomektomie | 36 | 49 | 45 | 31 | 30 | 18 | 9 | 9 | - | 9 | 16 |  |
|  |  | TUR-P | 303 | 241 | 350 | 267 | 246 | 206 | 175 | 155 | 225 | 266 | 243 |  |
|  |  | HoLEP |  |  |  | 1 | 10 | 12 | 37 | 87 | 104 | 8 | 119 |  |
|  |  | ThuLEP |  |  |  |  |  |  |  |  |  | 213 | 172 |  |
| Klinikum der Universität München (LMU Klinikum) | München | Chir. Adenomektomie | 24 | 22 | 28 | 29 | 18 | 1 | 1 | 1 | 1 | - | - |  |
|  |  | TUR-P | 254 | 308 | 287 | 259 | 285 | 219 | 247 | 205 | 205 | 178 | 151 |  |
|  |  | HoLEP |  |  |  |  | 83 | 209 | 175 | 229 | 303 | 380 | 490 |  |
|  |  | ThuLEP |  |  |  |  |  |  |  |  |  |  | - |  |
| Vivantes Auguste-Viktoria-Klinikum | Berlin | Chir. Adenomektomie | 1 | 1 | 1 | 1 | 4 | 1 | 6 | 6 | - | - | - |  |
|  |  | TUR-P | 418 | 244 | 584 | 165 | 149 | 172 | 197 | 166 | 127 | 82 | 95 |  |
|  |  | HoLEP |  |  |  | 645 | 562 | 617 | 834 | 819 | 1104 | 935 | 1146 |  |
|  |  | ThuLEP |  |  |  |  |  |  |  |  |  | 6 | 21 |  |

HoLEP=Holmium Laser Enukleation der Prostata, ThuLEP=Thulium Laser Enukleation der Prostata, TUR-P=Transurethrale Prostataresektion; chir.Adenomektomie: offene und laparoskopische (roboterassistierte) Verfahren
