# Supplementary material for: Contemporary surgical management of benign prostatic obstruction in Germany: A population-wide study based on German hospital quality report data from 2006 to 2019
Source: Urologe A. 2022 Feb 16;61(5):508–17. [Article in German] doi: 10.1007/s00120-022-01777-9 (PMC9072522; doi:10.1007/s00120-022-01777-9)
Supplement: Supplementary file 4 [file 120_2022_1777_MOESM4_ESM.docx]

**Abbildung B:**


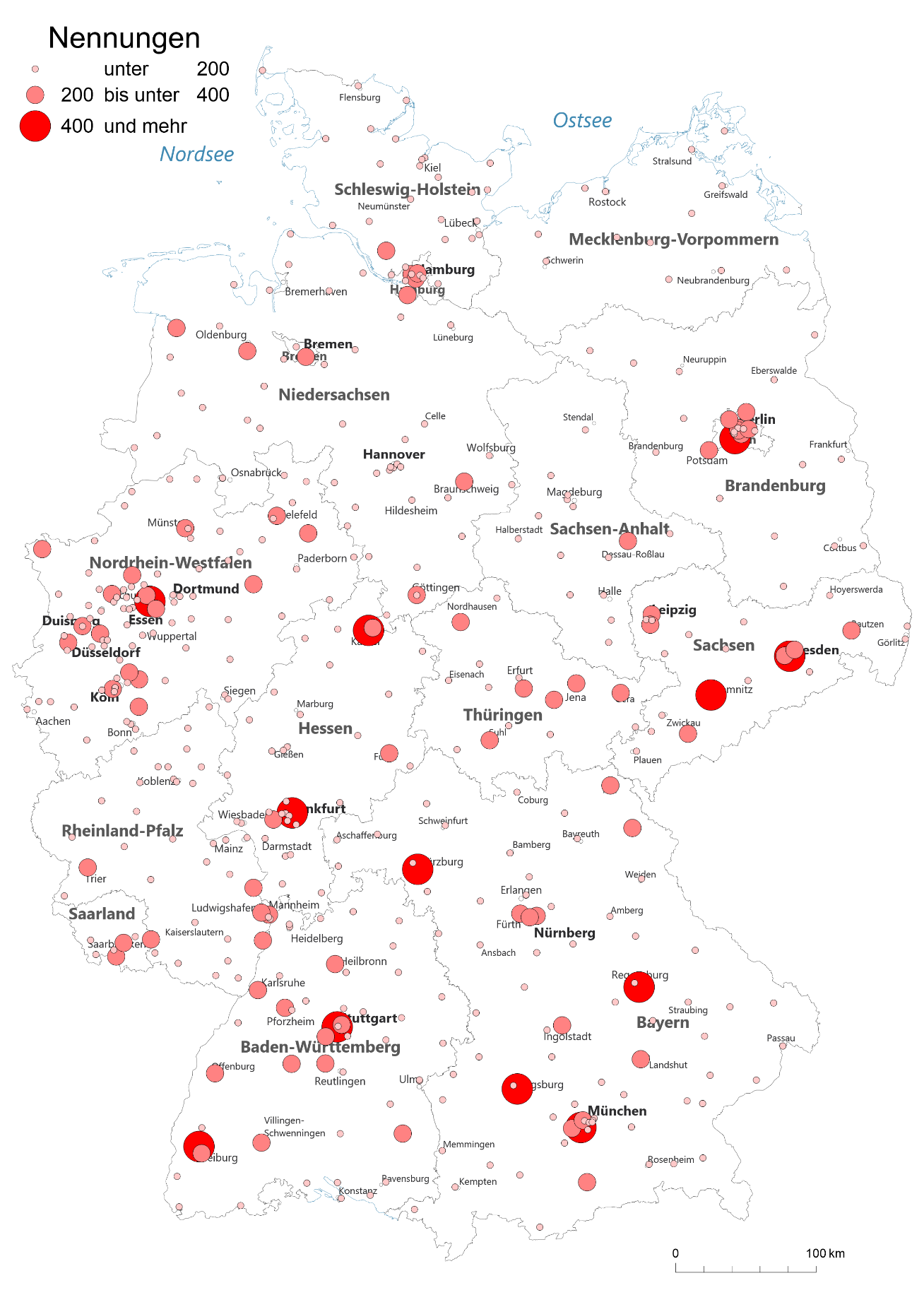


Abbildung B: Deutschlandweite Verteilung der BPH-Diagnosen im Jahr 2019 für urologische Fachabteilungen mit ≥100 Diagnosen. 2019 wurden insgesamt 64.581 stationäre BPH-Fälle von 472 urologischen Fachabteilungen dokumentiert. Dabei entfielen 97,2% auf den ICD-Code N40 und 2,8% auf D29.1.
